# Advanced age and differentiated thyroid carcinoma: a retrospective cohort study investigating surgical outcomes and recurrence risk

**DOI:** 10.3389/fendo.2026.1762393

**Published:** 2026-05-08

**Authors:** Eun Jin Kim, Seung Jae Lee, Eun Hwa Kim, Ji An Lee, Dong Hyun Seo, Jiahn Park, Sunmi Park, Sungkeun Kang, Sang Wook Kang, Jong Ju Jeong, Kee Hyun Nam, Woong Youn Chung, Young Suk Jo, Jandee Lee

**Affiliations:** 1 Department of Surgery, Open NBI Convergence Technology Research Laboratory, Yonsei Cancer Center, Severance Hospital, Yonsei University College of Medicine, Seoul, Republic of Korea; 2 Biostatistics Collaboration Unit, Department of Biomedical Systems Informatics, Yonsei University College of Medicine, Seoul, Republic of Korea; 3 Department of Internal Medicine, Open NBI Convergence Technology Research Laboratory, Yonsei University College of Medicine, Seoul, Republic of Korea

**Keywords:** age, age-related prognosis, differentiated thyroid cancer, older adults, recurrence, surgical complication

## Abstract

**Background:**

The increase in the older population globally presents unique challenges in thyroid cancer management. Age is a crucial prognostic factor in differentiated thyroid carcinoma (DTC); however, its effect on surgical outcomes in older patients is unclear. Therefore, we aimed to evaluate the clinical characteristics, postoperative complications, and long-term outcomes of DTC in older patients and determine the effect of advanced age on recurrence risk and surgical morbidity.

**Methods:**

A retrospective cohort study including 27,427 patients who underwent thyroidectomy for DTC between 2003 and 2019 was conducted. Patients were stratified into three age groups: Group A (16–64 years), Group B (65–74 years), and Group C (≥75 years). Clinicopathological characteristics, postoperative complications, and recurrence rates were analyzed. Multivariate Cox regression was performed to identify predictors of recurrence.

**Results:**

Older patients had more aggressive tumor features, including higher rates of extrathyroidal extension (Group C: 70.6% vs. Group A: 51.6%, p<0.0001) and distant metastasis (p=0.0006). Postoperative complications, including seroma (Group C: 15.58% vs. Group A: 2.85%, p<0.0001) and recurrent laryngeal nerve injury (Group C: 2.16% vs. Group A: 0.45%, p=0.0001), were more common in older patients. Recurrence risk increased with age (Group C: hazard ratio 1.894, 95% confidence interval: 1.156–3.104, p=0.0113). Despite higher risks, more extensive surgery was associated with improved survival in older patients.

**Conclusions:**

Advanced age is associated with more aggressive DTC and increased recurrence risk, yet thyroidectomy remains a viable treatment option. Close surveillance is essential to optimize outcomes in older patients.

## 1 Introduction

The global increase in the older population presents growing challenges in cancer care, particularly in tailoring treatment strategies that balance oncologic benefit with perioperative risk. In thyroid cancer, age is a well-established prognostic factor (1, 2). Accordingly, it is uniquely incorporated into the tumor-node-metastasis (TNM) staging system, in which 55 years is used as a cutoff to reflect its strong association with disease-specific outcomes (3–6). Despite this, differentiated thyroid carcinoma (DTC) including papillary, follicular, and oncocytic carcinoma, generally demonstrates an excellent prognosis, allowing for individualized treatment approaches based on patient characteristics and risk profiles (6–9).

In older patients, surgical decision-making is often influenced by concerns regarding comorbidities, reduced physiological reserve, polypharmacy, and the potential for perioperative complications (10–13). These concerns have historically led to more conservative management strategies in this population. However, longer life expectancy, along with advances in perioperative care and improvements in overall health and nutritional status, has resulted in an increasing number of older adults undergoing surgical intervention (3, 14, 15). Consequently, there is growing need for robust evidence evaluating the safety and effectiveness of surgical treatment specifically in older patients.

In particular, thyroid surgery is generally associated with shorter operative times and lower morbidity compared with operations for other solid malignancies (15–17). Nevertheless, several studies have reported that older patients may experience higher rates of postoperative complications, such as cervical hematoma and surgical site infection, potentially related to age-associated comorbidities, anticoagulant use, and impaired wound healing (18, 19). In addition, age-related nutritional and functional decline may adversely affect postoperative recovery and long-term outcomes (20, 21). Despite these concerns, data remain limited and inconsistent regarding whether advanced age independently impacts surgical morbidity and long-term oncologic outcomes in patients with DTC.

Therefore, this study aimed to analyze the clinical characteristics and postoperative outcomes of patients diagnosed with DTC categorized by age groups. We specifically compared surgical complication rates between adult and older adult groups and assessed age-related differences in disease characteristics and long-term outcomes, including recurrence. By focusing on DTC, this study seeks to provide clinically relevant evidence to guide appropriate surgical management and to avoid potential undertreatment of older patients with this increasingly prevalent malignancy.

## 2 Materials and methods

### 2.1 Study population

We retrospectively reviewed the medical records of patients aged ≥16 years who underwent thyroidectomy for DTC at Severance Hospital between January 2003 and June 2019. Among these patients, only those with DTC, including PTC, FTC, and OTC, were included. Patients diagnosed with DHGTC, PDTC, or anaplastic carcinoma were excluded. Patients with a follow-up period of less than 5 years or a history of malignancies other than DTC were also excluded. The need for obtaining written informed consent was waived because of the retrospective nature of the study. The study protocol was approved by the Institutional Review Board of Yonsei Cancer Center, Severance Hospital (IRB-No4–2024-1214), Seoul, Korea, and conducted according to the ethical standards of the Helsinki Declaration of 1975.

#### 2.1.2 Study design

Patients were categorized into three age groups (16–64, 65–74, and ≥75 years) based on clinically relevant geriatric thresholds. Age 65 years was used to define older adults, whereas age 75 years was selected to identify the oldest subgroup in whom physiologic vulnerability and perioperative risk may be more pronounced.

Clinicopathological data pertaining to patients, including sex, tumor size, histological type, extent of thyroid surgery, extent of lymph node dissection (LND), extrathyroidal extension (ETE), multifocality, and TNM stage, were collected. The histological classification of each tumor was based on the 2022 WHO classification, categorizing patients into PTC, FTC, or OTC (22). The TNM stage was determined according to the criteria of the American Joint Committee on Cancer staging system, 8^th^ edition (23).

The investigated surgical complications of thyroidectomy included hematoma, seroma, recurrent laryngeal nerve injury, and hypoparathyroidism. Recurrent laryngeal nerve injury was confirmed through fiberoptic laryngoscopy as unilateral or bilateral vocal cord paralysis and considered permanent if it persisted for more than 6 months. Hypoparathyroidism, defined as a serum parathyroid hormone level below 10 pg/mL, was classified as permanent if it persisted for more than 6 months postoperatively.

Blood tests and imaging studies, such as neck ultrasound or computed tomography, were performed in postoperative follow-up visits. Fine-needle aspiration biopsy was performed to confirm recurrence when it was suspected through imaging analysis.

The primary outcome of this study was long-term oncologic outcome, defined as recurrence-free survival and recurrence risk. Secondary outcomes included postoperative surgical complications, age-related differences in clinicopathological characteristics, extent of surgery, and cumulative recurrence rates at 5, 10, and 15 years.

### 2.2 Statistical analyses

Continuous variables that did not satisfy the normality assumption were analyzed using the Kruskal–Wallis test, and categorical variables were compared using the Chi-squared test. For the matched analyses, continuous variables were compared using linear mixed models, and categorical variables were analyzed using generalized estimating equations (GEE). Balance assessment using absolute standardized mean differences (ASMD) values was analyzed to evaluate post-matching balance. Univariate logistic regression followed by multivariate analysis was conducted to determine the association between age groups and surgical complications. Multivariate Cox regression analysis was performed to identify independent predictors of recurrence. A cumulative incidence plot was generated to assess the cumulative recurrence rate visually, and the log-rank test p-value was used to evaluate significant differences in cumulative incidence between groups. In addition, we conducted restricted follow-up analyses within clinically meaningful time horizons, restricted mean survival time (RMST) analyses, and landmark analyses including only patients surviving beyond predefined early time points. To control for confounding variables between age groups, a 1:5 matching was performed using sex and tumor size, which were identified as significant factors in the multivariate logistic regression analysis. Age groups were divided into two categories, with 65 years as the cutoff (**Supplementary Figure 1**). A p-value of <0.05 was considered statistically significant. SPSS Statistics software (Version 26.0; IBM, Armonk, NY, USA) or GraphPad Prism (GraphPad Software, San Diego, CA, USA) was used for statistical analyses.

## 3 Results

### 3.1 Patient demographics and baseline characteristics

Baseline characteristics of patients are summarized in **Table 1**. A total of 27,427 patients were enrolled, with 25,737 (93.8%) in Group A (aged 16–64 years), 1,459 (5.3%) in Group B (aged 65–74 years), and 231 (0.8%) in Group C (≥75 years). The mean age was 44 years in Group A, 68 years in Group B, and 77 years in Group C (p < 0.0001). Female predominance was consistent across groups, although a higher proportion of males was noted in Groups B and C. PTC was the predominant histologic subtype across all age groups; however, its prevalence showed a modest decline with advancing age, accompanied by a proportional increase in that of FTC and OTC (p < 0.0001). Histological analysis revealed age-related increases in overall ETE, including microscopic and gross ETE (Group A: 51.6%, Group B: 58.7%, Group C: 70.6%; p < 0.0001). Advanced T-stage tumors (T3/4) were also more prevalent in Group C (62.8%) than in Groups B (53.9%) and A (45.5%) (p < 0.0001). Similarly, the prevalence of N1b metastases was the highest in Group C (19.0%) compared to Groups B (9.9%) and A (9.3%). The extent of thyroidectomy and cervical LND also increased with age. Despite these differences, disease-free status was observed in 97.2% of patients in Group A, 95.2% of those in Group B, and 92.6% of those in Group C (p < 0.0001).

**Table 1:** Patient demographics and baseline clinical characteristics by age group.

Variables	Group A(N = 25,737)	Group B (N = 1,459)	Group C (N = 231)	*p*-value
Age, mean (years)	44 (36, 52)	68 (66, 70)	77 (75, 79)	<0.0001
Sex				0.0013
Male	4651 (18.1)	308 (21.1)	55 (23.8)	
Female	21086 (81.9)	1151 (78.9)	176 (76.2)	
Pathology				<0.0001
PTC	25472 (99)	1422 (97.5)	221 (95.7)	
FTC	243 (0.9)	29 (1.99)	9 (3.9)	
OTC	22 (0.1)	8 (0.6)	1 (0.4)	
Tumor size, mean (cm)	0.70 (0.50, 1.10)	0.80 (0.50, 1.30)	1.20 (0.70, 2.00)	<0.0001
Extrathyroidal extension				<0.0001
No	12470 (48.4)	602 (41.3)	68 (29.4)	
Microscopic	11930 (46.3)	769 (52.7)	145 (62.8)	
Gross	1337 (5.2)	88 (6.0)	18 (7.8)	
Multifocality				0.4104
Single	18071 (70.2)	1001 (68.6)	164 (71.0)	
Multifocality/bilateral	7666 (29.8)	458 (31.4)	67 (29.0)	
T stage				<0.0001
T1/2	14019 (54.5)	672 (46.1)	86 (37.2)	
T3/4	11718 (45.5)	787 (53.9)	145 (62.8)	
N stage				<0.0001
N0	15734 (61.1)	1006 (69.0)	151 (65.4)	
N1a	7603 (29.5)	308 (21.1)	36 (15.6)	
N1b	2400 (9.3)	145 (9.9)	44 (19.0)	
M stage				<0.0001
M0	25666 (99.7)	1445 (99.0)	224 (97.0)	
M1	71 (0.3)	14 (1.0)	7 (3.0)	
TNM stage				<0.0001
Stage I/II	19008 (73.8)	595 (40. 8)	94 (40.7)	
Stage III/IV	6729 (26.2)	864 (59.2)	137 (59.3)	
Extent of thyroidectomy				<0.0001
Lobectomy	13189 (51.2)	507 (34.8)	66 (28.6)	
Bilateral total thyroidectomy	12548 (48.8)	952 (65.2)	165 (71.4)	
Extent of cervical lymph node dissection				<0.0001
No	244 (1.0)	15 (1.0)	6 (2.6)	
Central compartment node dissection	22505 (87.4)	1255 (86.0)	177 (76.6)	
Modified radical neck dissection	2988 (11.6)	189 (13.0)	48 (20.8)	

Group A: age 16–64 years; Group B: age 65–74 years; Group C: age ≥75 years. PTC, papillary thyroid carcinoma; FTC, follicular thyroid carcinoma; OTC; oncocytic thyroid carcinoma.

To control for bias related to the extent of surgery, a subgroup analysis was conducted focusing exclusively on 13,665 patients who underwent bilateral total thyroidectomy (BTT). The analysis also demonstrated that with increasing age, the characteristics of thyroid cancer deteriorated, as evidenced by larger tumor size, higher rates of ETE, increased prevalence of T3/T4 and N1b metastasis, and higher recurrence rates (**Supplementary Table 1**). The extent of thyroidectomy and LND also increased with age.

### 3.2 Comparative analysis in matched cohorts

To mitigate the cofounding effects, a matched analysis was conducted. **Table 2** displays the outcomes of exact matching, where patients were divided into two groups based on the cutoff age of 65 years, with adjustments for sex and tumor size. Although the occurrence of ETE (16–64 years: 53.4%, ≥65 years: 60.4%, p < 0.0001) and M1 stage (16–64 years: 0.3%, ≥65 years: 1%, p = 0.0006) was significantly more common in advanced age groups, other histological factors including T-, N-, and TNM-stages were not significantly increased in older patients. Further, in this matched comparison, a lower percentage of older patients underwent extensive surgery, including BTT or modified radical neck dissection (MRND). This indicates that patients of the same sex and tumor size but advanced age tend to opt for less extensive surgery.

**Table 2:** Comparative analysis of clinical outcomes between two age groups in matched cohorts (N = 27,427).

Variables	Age 16–64 years (N = 5,010)	Age ≥65 years (N = 1,670)	*p*-value
Age, mean (years)	44.6 ± 10.2	69.6 ± 4	<0.0001
Sex			>0.999
Male	1074 (21.4)	358 (21.5)	
Female	3936 (78.6)	1312 (78.5)	
Pathology			<0.0001
PTC	4958 (99)	1631 (97.7)	
FTC	51 (1)	31 (1.9)	
OTC	1 (0)	8 (0.5)	
Tumor size, mean (cm)	1.1 ± 0.9	1.1 ± 0.9	>0.999
Extrathyroidal extension			<0.0001
No	2337 (46.7)	662 (39.6)	
Microscopic	2408 (48)	893 (53.5)	
Gross	265 (5.3)	115 (6.9)	
Multifocality			0.1529
Single	3352 (66.9)	1150 (68.9)	
Multifocality/bilateral	1658 (33.1)	520 (31.1)	
T stage			0.6842
T1/2	2299 (45.9)	757 (45.3)	
T3/4	2711 (54.1)	913 (54.7)	
N stage			<0.0001
N0	2921 (58.3)	1145 (68.6)	
N1a	1457 (29.1)	339 (20.3)	
N1b	632 (12.6)	186 (11.1)	
M stage			0.0006
M0	4993 (99.7)	1652 (98.9)	
M1	17 (0.3)	18 (1.1)	
TNM stage			<0.0001
Stage I/II	3293 (65.7)	686 (41.1)	
Stage III/IV	1717 (34.3)	984 (58.9)	
Extent of thyroidectomy			<0.0001
Lobectomy	1440 (28.7)	568 (34)	
Bilateral total thyroidectomy	3570 (71.3)	1102 (66)	
Extent of cervical lymph node dissection			<0.0001
No	95 (1.9)	21 (1.2)	
Central compartment node dissection	3952 (78.9)	1416 (84.8)	
Modified radical neck dissection	963 (19.2)	233 (14)	

PTC, papillary thyroid carcinoma; FTC, follicular thyroid carcinoma; OTC; oncocytic thyroid carcinoma. All values of absolute standardized mean differences (ASMD) for matched variables were less than 0.1.

After matching for sex and tumor size, patients who underwent BTT were categorized into two groups based on the cutoff age of 65 years (**Supplementary Table 2**). The prevalence of ETE and M1 stage was significantly greater in the advanced age group, but other histological factors, including T-, N-, and TNM-stage, were not significantly increased in older patients. Similar to the previous results, older patients underwent less extensive LND.

### 3.3 Postoperative complications

Postoperative complications varied significantly across age groups. The incidence of hematoma was higher in Group C (1.3%) than in Groups A (0.83%) and B (1.58%) (p = 0.0088). Although postoperative hematoma was more frequent in older patients, reoperation for hematoma evacuation did not differ significantly across age groups. Seroma formation was notably more frequent in Group C (15.58%) than in Groups A (2.85%) and B (9.25%) (p < 0.0001). Permanent recurrent laryngeal nerve injury was observed more frequently in Group C (2.16%) than in Groups A (0.45%) and B (0.82%) (p = 0.0001). However, the occurrence of permanent hypocalcemia was not significantly increased in older patients (**Supplementary Table 3**).

After adjusting for confounding factors, including sex, tumor size, pathological type, extent of surgery (thyroidectomy or LND), ETE, and TNM stage, the risk of surgical complications was compared among the three age groups (**Table 3**). Although hematoma was more frequent in older patients, the reoperation rate showed no significant difference. The incidence of wound seroma increased consistently with age (p < 0.001). The prevalence of permanent hoarseness was significantly increased in Group C (p = 0.0026).

**Table 3:** Multivariate logistic regression results of risk factors for postoperative complications.

Variables	Adjusted odds ratio (95% confidence interval)	*p*-value
Hematoma (n=27,427)		
Group A	1 (ref)	
Group B	1.787 (1.151, 2.775)	0.0097^$^
Group C	1.387 (0.477, 4.030)	0.5482^$^
Hematoma: Reoperation (n=27,427)		
Group A	1 (ref)	
Group B	0.909 (0.356, 2.320)	0.8424^$^
Group C	1.341 (0.273, 6.585)	0.7181^$^
Seroma (n=27,427)		
Group A	1 (ref)	
Group B	2.649 (2.167, 3.237)	<0.0001^$^
Group C	4.620 (3.165, 6.743)	<0.0001^$^
Transient hoarseness (n=27,427)		
Group A	1 (ref)	
Group B	1.068 (0.770, 1.482)	0.6945
Group C	0.779 (0.319, 1.905)	0.5841
Permanent hoarseness (Recurrent laryngeal nerve injury) (n=27,427)		
Group A	1 (ref)	
Group B	1.531 (0.843, 2.782)	0.1618^$^
Group C	3.749 (1.584, 8.873)	0.0026^$^
Transient hypocalcemia (n=13,665)		
Group A	1 (ref)	
Group B	0.711 (0.612, 0.825)	<0.0001
Group C	0.611 (0.428, 0.871)	0.0064
Permanent hypocalcemia (n=13,665)		
Group A	1 (ref)	
Group B	0.562 (0.345, 0.917)	0.0212^$^
Group C	0.532 (0.182, 1.558)	0.2497^$^

Group A: age 16–64 years; Group B: age 65–74 years; Group C: age ≥75 years. Adjusted variables (N = 27,427): sex, tumor size, pathology, extent of thyroidectomy, extent of cervical lymph node dissection, extrathyroidal extension, T stage 2gp, N stage, M stage, TNM stage. Adjusted variables (N = 13,665): sex, tumor size, pathology, extent of cervical lymph node dissection, extrathyroidal extension, T stage, N stage, M stage, TNM stage. $: Firth's bias correction.

### 3.4 Long-term clinical outcomes

Analysis of long-term outcomes revealed a higher recurrence rate in older patients. Descriptive analysis of recurrence patterns showed that locoregional recurrence was the predominant type across all age groups, whereas distant recurrence was infrequent. These descriptive data are presented in Supplementary Table 7. Disease-related mortality was rare across all age groups and was observed in 27 patients in Group A (0.1%), 5 patients in Group B (0.4%), and 2 patients in Group C (0.8%). The cumulative incidence of recurrence at 5 years was 1.7% in Group A, 2.7% in Group B, and 5.2% in Group C (p < 0.0001). At 10 years, recurrence rates increased to 3.0% in Group A, 5.1% in Group B, and 7.8% in Group C (**Supplementary Table 4**). Multiple Cox regression analysis identified advanced age as an independent predictor of recurrence, as the hazard ratio (HR) for recurrence was significantly higher in Group B (HR 1.577, 95% confidence interval [CI]: 1.209–2.058, p = 0.0008) and even higher in Group C (HR 1.894, 95% CI: 1.156–3.104, p = 0.0113) than in Group A. In addition, the recurrence rate was significantly higher in male patients and patients with FTC, OTC, ETE, higher N stage, and distant metastasis (**Table 4**). More aggressive surgical approaches (BTT or MRND) led to slightly better survival outcomes than lobectomy, particularly in Groups B and C.

**Table 4:** Multivariate Cox proportional hazards model for long-term outcomes.

Variables	Hazard ratio (95% confidence interval)	*p*-value
Age		
Group A	1 (ref)	
Group B	1.577 (1.209, 2.058)	0.0008
Group C	1.894 (1.156, 3.104)	0.0113
Sex		
Male	1 (ref)	
Female	0.843 (0.719, 0.989)	0.0355
Tumor size	1.109 (1.087, 1.131)	<0.0001
Pathology		
PTC	1 (ref)	
FTC	1.756 (1.150, 2.682)	0.0091
OTC	2.79 (0.877, 8.875)	0.0822
Extrathyroidal extension		
No	1 (ref)	
Yes	1.805 (1.407, 2.315)	<0.0001
T stage		
T1/2	1 (ref)	
T3/4	0.792 (0.623, 1.008)	0.0578
N stage		
N0	1 (ref)	
N1a	2.894 (2.428, 3.449)	<0.0001
N1b	7.439 (4.170, 13.273)	<0.0001
M stage		
M0	1 (ref)	
M1	23.1 (17.746, 30.07)	<0.0001
TNM stage		
Stage I/II	1 (ref)	
Stage III/IV	0.859 (0.726, 1.016)	0.0768
Extent of thyroidectomy		
Lobectomy	1 (ref)	
Bilateral total thyroidectomy	0.799 (0.670, 0.953)	0.0124
Extent of cervical lymph node dissection		
No	1 (ref)	
Central compartment node dissection	0.537 (0.274, 1.052)	0.0698
Modified radical neck dissection	0.378 (0.159, 0.901)	0.0282

Group A: age 16–64 years; Group B: age 65–74 years; Group C: age ≥75 years. PTC, papillary thyroid carcinoma; FTC, follicular thyroid carcinoma; OTC; oncocytic thyroid carcinoma.

**Figure 1:**
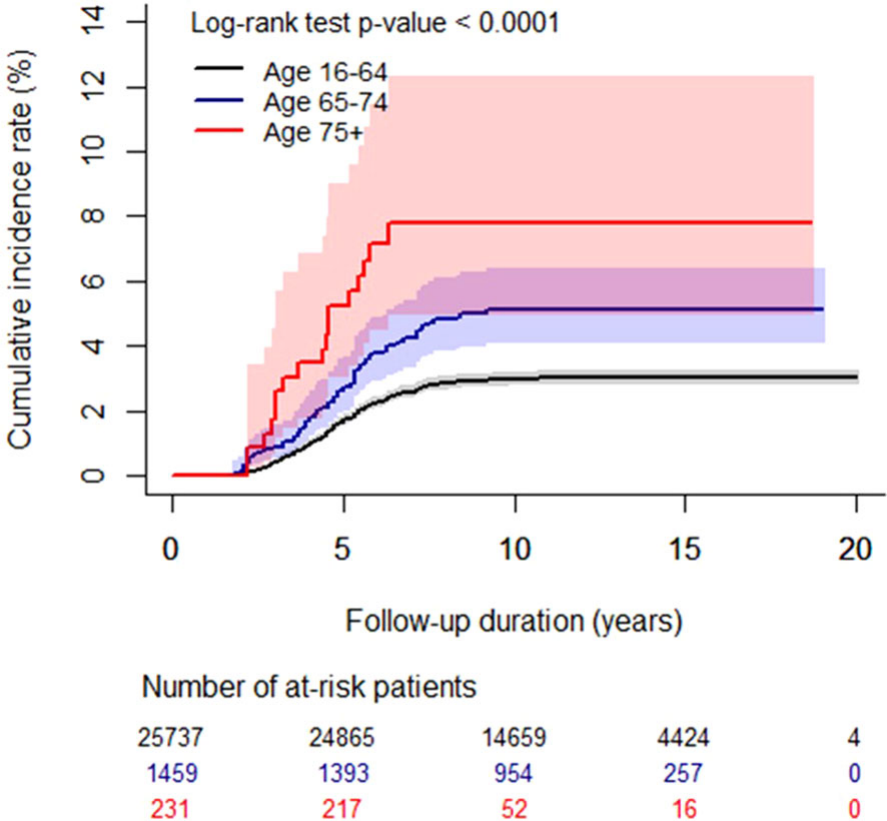
The cumulative incidence of recurrence stratified by age group: 16–64 years, 65–74 years, and 75+ years. The cumulative incidence rate (%) is shown over the follow-up period (years), with shaded areas indicating 95% confidence intervals. The number at risk represents the population remaining under observation without recurrence or censoring at each time point. The log-rank test demonstrated a statistically significant difference among the groups (p < 0.001).

**Table 5** displays the results of the comparison of the proportion of patients diagnosed with recurrence at 5, 10, and 15-year follow-up intervals. The recurrence rates significantly increased with age at each of these time points. Even after adjusting for factors such as sex, tumor size, pathology, ETE, and T-, N-, M-, and TNM stage, the recurrence rates increased with age. Furthermore, the plotted cumulative incidence indicated that the recurrence rates progressively increased with advancing age (**Figure 1**).

**Table 5:** Comparison of the 5-, 10-, 15- 15-year crude hazard ratios and adjusted hazard ratios for recurrence across age groups.

	Crude hazard ratio			Adjusted hazard ratio		
	Variables	HR (95% CI)	*p*-value	Variables	HR (95% CI)	*p*-value
5 years	Group A	1 (ref)		Group A	1 (ref)	
	Group B	1.584 (1.141, 2.197)	0.0059	Group B	1.450 (1.021, 2.059)	0.0059
	Group C	3.111 (1.753, 5.519)	0.0001	Group C	1.879 (1.040, 3.394)	0.0001
10 years	Group A	1 (ref)		Group A	1 (ref)	
	Group B	1.720 (1.346, 2.199)	<0.0001	Group B	1.582 (1.212, 2.065)	<0.0001
	Group C	2.994 (1.851, 4.844)	<0.0001	Group C	1.894 (1.156, 3.105)	<0.0001
15 years	Group A	1 (ref)		Group A	1 (ref)	
	Group B	1.710 (1.338, 2.186)	<0.0001	Group B	1.577 (1.209, 2.058)	<0.0001
	Group C	2.986 (1.846, 4.830)	<0.0001	Group C	1.894 (1.156, 3.104)	<0.0001

Group A: age 16–64 years; Group B: age 65–74 years; Group C: age ≥75 years. * Adjusted variables sex, tumor size, pathology, extent of cervical lymph node dissection, extrathyroidal extension, T stage, N stage, M stage, TNM stage. HR, hazard ratio; CI, confidence interval.

## 4 Discussion

This study provides a comprehensive analysis of the clinical characteristics, postoperative complications, and long-term outcomes of DTC in older patients. Our findings demonstrate that increasing age was associated with more aggressive disease features, higher recurrence rates, and an increased risk of surgical complications. Despite these challenges, the data suggest that when selected carefully, thyroidectomy remains a viable and beneficial treatment option for older patients.

A key finding of this study is the association between advanced age and more aggressive tumor characteristics. Older patients exhibited higher rates of ETE, advanced T-stage, N1b metastases, M stage, and TNM stage compared to younger patients (**Table 1**). These findings align with those of previous studies, suggesting that thyroid cancer is associated with a more aggressive biological behavior in older patients (24, 25). However, after adjusting for sex and tumor size, both N1a and N1b metastases were more prevalent in younger patients (**Table 2**). Some studies have found a higher prevalence of LN metastasis in younger patients (26, 27), while others have observed a U-shaped pattern, with greater prevalence in both the youngest and oldest age groups (28). In our study, the discrepancy between the unmatched and matched analyses indicates that tumor size substantially mediates the relationship between age and nodal status. In the unmatched cohort, older patients presented with larger tumors and more advanced stage disease, which may partly reflect delayed diagnosis or differences in healthcare utilization. When tumor size was controlled for, the age-related difference in lymph node metastasis diminished or reversed, indicating that nodal involvement may be strongly influenced by tumor burden at presentation rather than age alone. Therefore, while older patients exhibited higher rates of extrathyroidal extension, distant metastasis, and advanced TNM stage, the pattern of lymph node metastasis appears to be more closely related to tumor size and detection timing. These findings suggest that the observed aggressiveness in older patients may reflect a combination of biological factors and stage migration rather than uniformly increased metastatic potential.

The matched cohort analysis provides additional insight into the effect of age on surgical decision-making. Notably, even after matching for tumor size and sex, older patients were significantly less likely to undergo BTT or MRND (**Table 2**). This suggests that age itself may influence the extent of surgical management, potentially reflecting concerns regarding comorbidities, frailty, or perioperative risk. Importantly, in the multivariable Cox regression analysis, BTT was associated with a lower recurrence risk compared with lobectomy (HR 0.799, p = 0.0124), and MRND was associated with a substantially reduced recurrence risk compared with no or central lymph node dissection (HR 0.378, p = 0.0282). These findings indicate that, in appropriately selected patients, more extensive surgical approaches are associated with improved oncologic outcomes (**Table 4**). When interpreted together, these results suggest a potential gap between oncologic benefit and surgical practice in older patients. Although more extensive surgery appears to be associated with reduced recurrence risk, older individuals were less likely to receive such procedures. While residual confounding and treatment selection bias cannot be excluded due to the observational nature of this study, these findings underscore the importance of individualized surgical decision-making rather than routine de-escalation based solely on chronological age.

Our study also highlights the increased risk of postoperative complications in older patients undergoing thyroidectomy. Specifically, seroma formation and permanent hoarseness were significantly more common in older individuals. These findings are consistent with those of previous reports, indicating that older patients have a reduced physiological reserve and are more susceptible to surgical morbidity (29, 30). However, despite increased complication rates, reoperation rates for hematoma were not significantly different across age groups, suggesting that while older patients may have a higher risk of minor postoperative complications, severe complications requiring additional surgical intervention remained relatively uncommon. The occurrence of transient hypoparathyroidism, which is associated with the extent of surgery (lobectomy vs. BTT), was more common in younger patients. This finding is consistent with that of previous studies showing a tendency for more extensive surgeries to be performed in younger patients with thyroid cancer (25, 31). While complications due to comorbidities may increase in older patients, advanced age itself does not appear to significantly increase the risks associated with surgery alone (32–35).

Long-term outcomes further illustrate the adverse impact of advanced age on disease recurrence. Our study identified age as an independent predictor of recurrence, with a significantly higher recurrence rate in older patients even after adjusting for potential confounders such as sex, tumor size, and TNM stage. These results corroborate the findings of prior studies demonstrating an increased risk of disease recurrence and disease-specific mortality in older patients with thyroid cancer (17, 36). The reasons for this increased recurrence risk remain unclear but may be related to delayed diagnosis, altered tumor biology, or a less aggressive treatment approach in older patients (37–39). Our findings suggest that while thyroid cancer remains a highly treatable disease, older patients require close surveillance due to the higher likelihood of disease recurrence over time.

To further examine whether this association could be influenced by differential survivor duration across age groups, we performed RMST analyses. In the RMST analysis, recurrence-free survival progressively decreased with advancing age. At 10 years, the oldest group exhibited a 3.6-month shorter recurrence-free duration compared with younger patients, and this difference widened over 6 months at 15 years. These findings suggest that the age-associated difference in recurrence is not solely attributable to imbalance in long-term follow-up (**Supplementary Table 5**). In addition, we conducted landmark analyses including only patients who survived beyond predefined early time points (**Supplementary Table 6**). This approach reduces potential bias arising from higher early mortality in the oldest age group. The results of the landmark analyses were directionally consistent with those of the primary Cox regression model, further supporting the robustness of the association between advanced age and recurrence risk.

Some limitations should be considered when interpreting our findings. First, the retrospective nature of this study introduces potential selection bias, and although we employed rigorous statistical adjustments, several unadjusted confounders may have influenced our results. Second, because only surgically treated patients were included, our cohort represents a selected population of older adults deemed fit for operative management. Therefore, the generalizability of our findings to frail elderly patients or those managed non-operatively may be limited. Third, although complementary analyses including restricted follow-up intervals, RMST, and landmark approaches were performed to mitigate potential bias related to differential survival, formal competing mortality cannot be completely excluded. Fourth, although our institution uses a postoperative surveillance protocol, we must consider the potential impact of differences in follow-up intensity or standardized detection rates of recurrence across age groups. Fifth, RAI avidity and formal response classification could not be reliably analyzed because these data were not uniformly available in a standardized format across the full retrospective cohort, partly owing to referral of postoperative RAI treatment to outside institutions during earlier years of the study period. Finally, the relative small sample size in the ≥75-year group may have limited statistical power for certain subgroup analyses, particularly for less frequent outcomes such as distant metastasis and permanent complications. Larger multicenter studies are warranted to validate our findings in this population.

This study underscores the impact of advanced age on the clinical presentation, postoperative complications, and long-term outcomes of patients with DTC. Older patients exhibit more aggressive tumor features and a higher recurrence risk, necessitating careful postoperative surveillance. While older individuals are more prone to minor surgical complications, surgery is a feasible treatment option, particularly when tailored to patients’ overall health status. Further research is warranted to optimize treatment strategies and improve outcomes in this growing patient population.

## Data Availability

The datasets generated during and/or analyzed during this study are available from the corresponding author on request.
