# Supplementary material for: Advanced age and differentiated thyroid carcinoma: a retrospective cohort study investigating surgical outcomes and recurrence risk
Source: Front Endocrinol (Lausanne). 2026 May 8;17:1762393. doi: 10.3389/fendo.2026.1762393 (PMC13193919; doi:10.3389/fendo.2026.1762393)
Supplement: Supplementary file 1 [file DataSheet1.pdf]

Supplementary Material

Advanced Age and Differentiated Thyroid Carcinoma: A Retrospective Cohort Study Investigating Surgical Outcomes and Recurrence Risk

Eun Jin Kim[1†], Seung Jae Lee[1†], Eun Hwa Kim[2], Ji An Lee[1], Dong Hyun Seo[3], Jiahn Park[1], Sunmi Park[3], Sungkeun Kang[1], Sang Wook Kang[1], Jong Ju Jeong[1], Kee Hyun Nam[1], Woong Youn Chung[1], Young Suk Jo[3]*, and Jandee Lee[1]*

*** Correspondence:** Jandee Lee: jandee@yuhs.ac; Young Suk Jo: joys@yuhs.ac

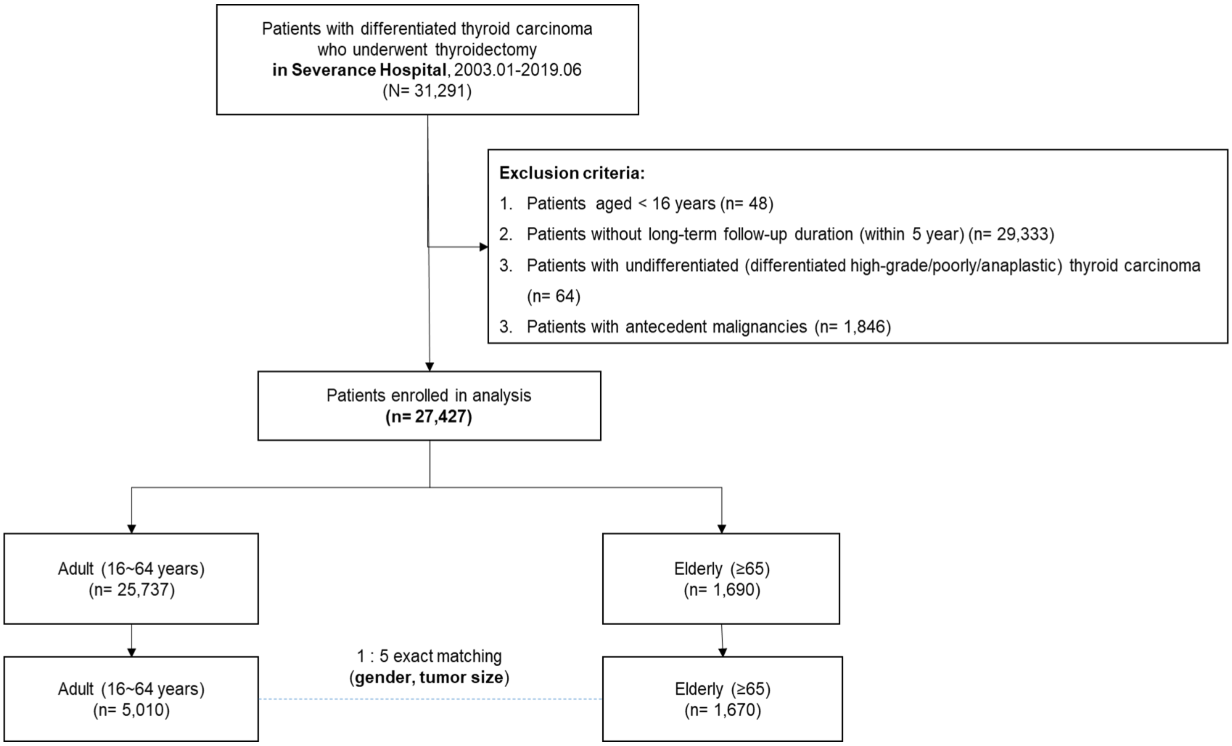

Supplementary Figure 1. Schematic flow of study design

Supplementary Table 1. **Clinical and pathological characteristics in bilateral total thyroidectomy cases (N=13,665)**

Variables	Group A (N=12,548)	Group B (N=952)	Group C (N=165)	p-value
**Age, mean (years)**	46 (37, 53)	68 (66, 70)	77 (75, 79)	<0.0001
**Sex**				0.1387
Male	2,263 (18)	189 (19.9)	37 (22.4)	
Female	10,285 (82)	763 (80.1)	128 (77.6)	
**Pathology**				<0.0001
PTC	12,479 (99.5)	932 (97.9)	161 (97.6)	
FTC	64 (0.5)	18 (1.9)	4 (2.4)	
OTC	5 (0)	2 (0.2)	0 (0)	
**Tumor size, mean (cm)**	0.9 (0.7, 1.4)	0.9 (0.6, 1.5)	1.3 (0.9, 2.1)	<0.0001
**Extrathyroidal extension**				0.0011
No	4,495 (35.8)	313 (32.9)	39 (23.6)	
Microscopic	7,388 (58.9)	575 (60.4)	112 (67.9)	
Gross	665 (5.3)	64 (6.7)	14 (8.5)	
**Multifocality**				0.6169
Single	8,793 (70.1)	658 (69.1)	111 (67.3)	
Multifocality/bilateral	3,755 (29.9)	294 (30.9)	54 (32.7)	
**T stage**				<0.0001
T1/2	4,933 (39.3)	330 (34.7)	43 (26.1)	
T3/4	7,615 (60.7)	622 (65.3)	122 (73.9)	
**N stage**				<0.0001
N0	6,010 (47.9)	580 (60.9)	95 (57.6)	
N1a	4,170 (33.2)	227 (23.8)	26 (15.8)	
N1b	2,368 (18.)	145 (15.2)	44 (26.6)	
**M stage**				<0.0001
M0	12,481 (99.5)	938 (98.5)	158 (95.8)	
M1	67 (0.5)	14 (1.5)	7 (4.2)	
**TNM stage**				<0.0001
Stage I/II	7,940 (63.3)	273 (28.7)	50 (30.3)	
Stage III/IV	4,608 (36.7)	679 (71.3)	115 (69.7)	
**Extent of cervical lymph node dissection**				0.0035

No	33 (0.3)	2 (0.2)	2 (1.2)	
Central compartment node dissection	9,701 (77.3)	769 (80.8)	115 (69.7)	
Modified radical neck dissection	2,814 (22.4)	181 (19)	48 (29.1)	
**Recurrence**				<0.0001
Disease free	12,113 (96.5)	892 (93.7)	150 (90.9)	
Recurrence	435 (3.5)	60 (6.3)	15 (9.1)	

Group A: age 16–64 years; Group B: age 65–74 years; Group C: age ≥75 years

PTC, papillary thyroid carcinoma; FTC, follicular thyroid carcinoma; OTC; oncocytic thyroid carcinoma

Supplementary Table 2. Clinical and pathological characteristics in bilateral total thyroidectomy cases in matched cohorts (N=13,665)

Variables	Age 16–64 years (N=3,273)	Age ≥65 years (N=1,091)	p-value
**Age, mean (years)**	45.2 ± 10.3	69.7 ± 4.1	<0.0001
**Sex**			>0.999
Male	666 (20.3)	222 (20.4)	
Female	2607 (79.7)	869 (79.6)	
**Pathology**			<0.0001
PTC	3261 (99.6)	1073 (98.3)	
FTC	12 (0.4)	17 (1.6)	
OTC	0	1 (0.1)	
**Tumor size, mean (cm)**	1.2 ± 0.8	1.2 ± 0.84	>0.999
**Extrathyroidal extension**			<0.0001
No	1249 (38.2)	341 (31.3)	
Microscopic	1854 (56.6)	682 (62.5)	
Gross	170 (5.2)	68 (6.2)	
**Multifocality**			0.3210
Single	2195 (67.1)	749 (68.7)	
Multifocality/bilateral	1078 (32.9)	342 (31.3)	
**T stage**			0.0250
T1/2	1236 (37.8)	372 (34.1)	
T3/4	2037 (62.2)	719 (65.9)	
**N stage**			<0.0001
N0	1646 (50.3)	665 (61)	
N1a	1044 (31.9)	246 (22.5)	
N1b	583 (17.8)	180 (16.5)	
**M stage**			0.0009
M0	3257 (99.5)	1074 (98.4)	
M1	16 (0.5)	17 (1.6)	
**TNM stage**			<0.0001
Stage I/II	1947 (59.5)	320 (29.3)	
Stage III/IV	1326 (40.5)	771 (70.7)	

**Extent of cervical lymph node dissection**			<0.0001
No	15 (0.5)	3 (0.3)	
Central compartment node dissection	2390 (73)	868 (79.6)	
Modified radical neck dissection	868 (26.5)	220 (20.1)	
**Recurrence**			0.1705
Disease free	3112 (95.1)	1024 (93.9)	
Recurrence	161 (4.9)	67 (6.1)	

PTC, papillary thyroid carcinoma; FTC, follicular thyroid carcinoma; OTC; oncocytic thyroid carcinoma

Supplementary Table 3. Postoperative complications stratified by age group

Variables	Group A (N=25,737)	Group B (N=1,459)	Group C (N=231)	p-value
**Postoperative hematoma**				0.0088
(-)	25524 (99.2)	1436 (98.4)	228 (98.7)	
(+)	213 (0.8)	23 (1.6)	3 (1.3)	
**Reoperation for postoperative hematoma**				0.7312
(-)	25659 (99.7)	1455 (99.7)	230 (99.6)	
(+)	78 (0.3)	4 (0.3)	1 (0.4)	
**Seroma**				<0.0001
(-)	25004 (97.2)	1324 (90.8)	195 (84.4)	
(+)	733 (2.8)	135 (9.2)	36 (15.6)	
**Transient hoarseness**				0.8018
(-)	25071 (97.4)	1418 (97.2)	226 (97.8)	
(+)	666 (2.6)	41 (2.8)	5 (2.2)	
**Permanent hoarseness (Recurrent laryngeal nerve injury)**				0.0001
(-)	25622 (99.6)	1447 (99.2)	226 (97.8)	
(+)	115 (0.5)	12 (0.8)	5 (2.2)	
**Transient hypocalcemia (n=13,665)**				<0.0001
(-)	7991 (63.7)	675 (70.9)	121 (73.3)	
(+)	4557 (36.3)	277 (29.1)	44 (26.7)	
**Permanent hypocalcemia (n=13,665)**				0.1015
(-)	12185 (97.1)	935 (98.2)	162 (98.2)	
(+)	363 (2.9)	17 (1.8)	3 (1.8)	

Group A: age 16–64 years; Group B: age 65–74 years; Group C: age ≥75 years

All return-to-operating-room events in this cohort were reoperations for postoperative hematoma evacuation.

Supplementary Table 4. Cumulative incidence rate for recurrence across age groups

	Cumulative incidence rate			
	**Group A**	**Group B**	**Group C**	**Log-rank test p-value**
**5 years**	0.017 (0.016, 0.019)	0.027 (0.020, 0.037)	0.052 (0.030, 0.090)	<0.0001
**10 years**	0.030 (0.028, 0.032)	0.051 (0.041, 0.064)	0.078 (0.049, 0.123)	<0.0001
**15 years**	0.032 (0.022, 0.034)	0.059 (0.065, 0.087)	0.082 (0.054, 0.157)	<0.0001

Group A: age 16–64 years; Group B: age 65–74 years; Group C: age ≥75 years

Supplementary Table 5. Restricted mean survival time (RMST) by age group

	Group A	Group B	Group C	A vs. B	B vs. C	C vs. A
**5 years**	59.741 (59.711, 59.77)	59.521 (59.342, 59.70)	59.004 (58.374, 59.634)	-0.220 (-0.401, -0.039) p-value = 0.017	-0.736 (-1.367, -0.106) p-value = 0.022	-0.516 (-1.171, 0.139) p-value = 0.122
**10 years**	118.177 (118.041, 118.313)	116.889 (116.146, 117.631)	114.532 (111.997, 117.068)	-1.288 (-2.043, -0.533) p-value = 0.001	-3.645 (-6.184, -1.106) p-value = 0.005	-2.356 (-4.998, 0.286) p-value = 0.080
**15 years**	176.374 (176.113, 176.635)	173.827 (172.407, 175.247)	169.841 (165.180, 174.503)	-2.547 (-3.991, -1.103) p-value = 0.001	-6.532 (-11.202, -1.863) p-value = 0.006	-3.985 (-8.859, 0.888) p-value = 0.109

Group A: age 16–64 years; Group B: age 65–74 years; Group C: age ≥75 years.

RMST standardized reporting order (time horizon → RMST by group → between-group difference with 95% CI and p-value).

Supplement Table 6. Landmark Cox regression analysis of Recurrence Stratified by Age Group

Landmark Time	Follow-up Duration	Crude hazard ratio			Adjusted hazard ratio		
		Variables	HR (95% CI)	*p*-value	Variables	HR (95% CI)	*p*-value
**3years**	2 years	Group A	1 (ref)		Group A	1 (ref)	
		Group B	1.403 (0.942, 2.092)	0.0961	Group B	1.232 (0.805, 1.885)	0.3375
		Group C	2.075 (0.926, 4.653)	0.0763	Group C	1.250 (0.548, 2.847)	0.5961
	7 years	Group A	1 (ref)		Group A	1 (ref)	
		Group B	1.648 (1.256, 2.162)	0.0003	Group B	1.478 (1.101, 1.984)	0.0094
		Group C	2.338 (1.287, 4.244)	0.0053	Group C	1.559 (0.849, 2.862)	0.1523
	12 years	Group A	1 (ref)		Group A	1 (ref)	
		Group B	1.637 (1.248, 2.147)	0.0004	Group B	1.472 (1.097, 1.977)	0.0100
		Group C	2.330 (1.283, 4.230)	0.0054	Group C	1.559 (0.849, 2.862)	0.1521
	Total duration	Group A	1 (ref)		Group A	1 (ref)	
		Group B	1.637 (1.248, 2.147)	0.0004	Group B	1.472 (1.097, 1.977)	0.0100
		Group C	2.330 (1.283, 4.230)	0.0054	Group C	1.559 (0.849, 2.862)	0.1521
**5year**	5 years	Group A	1 (ref)		Group A	1 (ref)	
		Group B	1.930 (1.332, 2.797)	0.0005	Group B	1.801 (1.195, 2.713)	0.0049
		Group C	2.739 (1.131, 6.635)	0.0256	Group C	2.518 (1.025, 6.184)	0.0439
	10 years	Group A	1 (ref)		Group A	1 (ref)	
		Group B	1.902 (1.313, 2.756)	0.0007	Group B	1.786 (1.186, 2.688)	0.0055
		Group C	2.716 (1.122, 6.578)	0.0268	Group C	2.517 (1.025, 6.179)	0.0440
	Total duration	Group A	1 (ref)		Group A	1 (ref)	
		Group B	1.902 (1.313, 2.756)	0.0007	Group B	1.786 (1.186, 2.688)	0.0055
		Group C	2.716 (1.122, 6.578)	0.0268	Group C	2.517 (1.025, 6.179)	0.0440

Group A: age 16–64 years; Group B: age 65–74 years; Group C: age ≥75 years.

* Adjusted variables: sex, tumor size, pathology, extent of cervical lymph node dissection, extrathyroidal extension, T stage, N stage, M stage, TNM stage

HR, hazard ratio; CI, confidence interval

Supplementary Table 7. Pattern of Recurrence according to Age Group

Age Group	Recurrence category	Site of recurrence	Number of patients (%)
Group A	Local	Contralateral thyroid	253
		Thyroid bed	73
	Nodal	Central or Lateral neck node	358
		Subtotal	684 (94.3)
	Distant	Lung	23
		Bone	6
		Lung and Bone	12
		Subtotal	41 (5.7)
Group B	Local	Contralateral thyroid	21
		Thyroid bed	7
	Nodal	Central or Lateral neck node	30
		Subtotal	58 (82.9)
	Distant	Lung	8
		Bone	1
		Lung and Bone	3
		Subtotal	12 (17.1)
Group C	Local	Contralateral thyroid	5
		Thyroid bed	1
	Nodal	Central or Lateral neck node	9
		Subtotal	15 (88.2)
	Distant	Lung	1
		Bone	0
		Lung and Bone	1
		Subtotal	2 (11.8)

Group A: age 16–64 years; Group B: age 65–74 years; Group C: age ≥75 years.

Percentages were calculated using the number of patients with recurrence in each age group as the denominator.
